# Critical Requirements for the Initiation of a Cardiac Arrhythmia in Rat Ventricle: How Many Myocytes?

**DOI:** 10.3390/cells11121878

**Published:** 2022-06-09

**Authors:** Aman Ullah, Minh Tuan Hoang-Trong, William Jonathan Lederer, Raimond L. Winslow, Mohsin Saleet Jafri

**Affiliations:** 1 School of Systems Biology, Krasnow Institute for Advanced Study, George Mason University, Fairfax, VA 22030, USA; aullah3@gmu.edu (A.U.); hoangtrongminhtuan@gmail.com (M.T.H.-T.); 2 Biomedical Engineering and Technology, University of Maryland School of Medicine, Baltimore, MD 21201, USA; jlederer@umaryland.edu; 3 Institute for Computational Medicine and Department of Biomedical Engineering, The Johns Hopkins University, Baltimore, MD 20218, USA; r.winslow@northeastern.edu; 4 The Roux Institute, Northeastern University, Portland, ME 04102, USA

**Keywords:** ventricular myocyte network, computational model, heart failure, arrhythmia

## Abstract

Cardiovascular disease is the leading cause of death worldwide due in a large part to arrhythmia. In order to understand how calcium dynamics play a role in arrhythmogenesis, normal and dysfunctional Ca^2+^ signaling in a subcellular, cellular, and tissued level is examined using cardiac ventricular myocytes at a high temporal and spatial resolution using multiscale computational modeling. Ca^2+^ sparks underlie normal excitation–contraction coupling. However, under pathological conditions, Ca^2+^ sparks can combine to form Ca^2+^ waves. These propagating elevations of (Ca^2+^)_i_ can activate an inward Na^+^–Ca^2+^ exchanger current (I_NCX_) that contributes to early after-depolarization (EADs) and delayed after-depolarizations (DADs). However, how cellular currents lead to full depolarization of the myocardium and how they initiate extra systoles is still not fully understood. This study explores how many myocytes must be entrained to initiate arrhythmogenic depolarizations in biophysically detailed computational models. The model presented here suggests that only a small number of myocytes must activate in order to trigger an arrhythmogenic propagating action potential. These conditions were examined in 1-D, 2-D, and 3-D considering heart geometry. The depolarization of only a few hundred ventricular myocytes is required to trigger an ectopic depolarization. The number decreases under disease conditions such as heart failure. Furthermore, in geometrically restricted parts of the heart such as the thin muscle strands found in the trabeculae and papillary muscle, the number of cells needed to trigger a propagating depolarization falls even further to less than ten myocytes.

## 1. Introduction

Ill-timed propagated ectopic beats can initiate a potentially fatal arrhythmia, with the most serious being ventricular fibrillation [1,2]. Both atrial and ventricular fibrillation are a rapidly growing global health problem mainly due to aging of the human population, diseases such as heart failure, the adoption of unhealthy lifestyles, and channelopathies and other genetic factors in younger patient populations [3,4]. Despite efforts by clinicians and scientists, cardiac arrhythmia remains a significant health problem accounting for 15–20% of all deaths [5]. Previous studies have demonstrated that aberrations in Ca^2+^ dynamics can play a role in cardiac arrhythmia [6,7,8,9,10]. These focal excitations in an individual myocyte can be caused by spontaneous Ca^2+^ release resulting in Ca^2+^ waves. A Ca^2+^ wave in a ventricular myocyte is initiated when spontaneous Ca^2+^ is released from the sarcoplasmic reticulum (SR) via ryanodine receptors ((RyR2)/Ca^2+^ release channels) and diffuses to adjacent release sites (dyads) where they trigger additional Ca^2+^ release. The spread of this process to different release sites results in a Ca^2+^ wave. The rapid change of Ca^2+^ results in a depolarization inward current through the Na^+^–Ca^2+^ exchanger (I_NCX_), which depolarizes the cell membrane sufficiently to trigger a beat, as demonstrated by previous experiments and computational modeling [6,11,12,13].

When an action potential (AP) travels through cardiac tissue, the wavefront acts as a source of depolarizing current for the repolarized myocytes nearby (the sink). The source current density must be high enough to activate the sink, and if the source–sink mismatch is too significant, propagation will fail [14]. The propagating wavefronts of an AP have been studied extensively previously. Some earlier studies that have investigated this question suggest that as many as approximately ~700,000 myocytes must undergo such behavior to initiate a propagating action potential or an arrhythmia in a block of heart tissue [14,15]. The development of successful, empirical methods for the treatment of cardiac arrhythmias is possibly only through knowledge of the underlying biology [16]. This paper addresses the basic questions of how many myocytes are needed to trigger cardiac arrhythmia in 1-D, 2-D, and 3-D tissue. Simulations in 1-D, 2-D, and 3-D represent physical structures in the heart (see [17] for more details). Simulations in 1D can represent fiber strands such as Purkinje or cardiac trabeculae [18]. Simulations in 2-D represent myocardia sheets reflecting that the heart is arranged in fiber sheets with the conductivity in the sheet being greater than between the sheets. The 3-D simulations can represent blocks of heart tissue such as the ventricular wall.

In order to understand how Ca^2+^ dynamics plays a role in arrhythmogenesis, model simulations explore normal and dysfunctional Ca^2+^ signaling in cardiac ventricular myocytes at a high temporal and spatial resolution. These findings suggest processes that lead to the initiation of an arrhythmia by current injection and spontaneous Ca^2+^ release during heart failure. The goal of this study is to achieve a realistic and adequate quantitative understanding of the cardiac arrhythmias, i.e., the number of myocytes needed to trigger a sustained depolarization in a tissue. The model describes the most elementary event of cardiac Ca^2+^ release, the calcium spark, with a stochastic model that explains the mechanisms of Ca^2+^ release termination, graded Ca^2+^ release, Ca^2+^ homeostasis, and the sarcoplasmic reticulum calcium leak, and the generation of arrhythmias from defects in Ca^2+^ signaling.

The computational modeling of cardiac arrhythmias presented here provided a new understanding of Ca^2+^-entrained arrhythmias and suggest that the aberrant activation of only a small number of myocytes is required to generate an arrhythmia. The model also suggests the role of recruitment of Ca^2+^ release sites play in the initiation of arrhythmias.

## 2. Materials and Methods

### 2.1. Model

This study integrated our previous local-control model of excitation–contraction coupling rat ventricular myocytes into a network of myocytes to describe ventricular tissue [11,19]. Local-control is the feature that excitation–contraction coupling is modulated locally at the release site level. This model features the fully stochastic gating of ion channels from 20,000 calcium release sites using the algorithm developed in the previous paper [20]. Each release site contains a cluster of 49 RyR2 channels and a smaller cluster of 7 L-type calcium channels in closed proximity to each other, forming the dyadic subspace known as calcium release units (CRUs).

The model used in this paper has several important features, some of which were introduced in [11,19,20,21], such as a spark model well-constrained by experimental data and a thermodynamically and biochemically constrained model for SERCA2a (sarco- and endoplasmic reticulum Ca^2+^ ATPase isoform 2a) pump [22]. Other changes include using (1) explicit buffer dynamics in the subspace, (2) a different L-type Ca^2+^ channel that incorporate Ca^2+^-bound calmodulin-dependent inactivation and updated parameters based on newer experimental data from rat ventricular myocyte [23]. In addition, by using the novel computational method and GPGPU (general purpose graphical processing unit) technology, it now allows us to do larger scale simulations that provide insights into calcium dynamics.

The current Ca^2+^ spark model was developed using the mean-field energetic coupling between RyR2s distributed in an array that has been observed in biological systems [24,25]. The RyR2 model, which features both calcium-dependent activation from dyadic cleft Ca^2+^ and RyR2 open probability dependence on SR lumen Ca^2+^, can explain the robust mechanism of spark initiation and spark termination [26]. The model has been used to explain the diastolic Ca^2+^ leak with how stochastic gating of RyR2s and a thermodynamic SERCA2a model can balance SERCA2a-pump activity at the quiescent condition [20]. Recently, experiments have shown that dyad models with RyR2 gating without Ca^2+^-dependent inactivation produce more realistic Ca^2+^ spark results than those that assumed [27]. By increasing SR buffer capacity, it increases Ca^2+^ spark, which supports the idea that depletion of SR release plays a more dominant role in spark termination than Ca^2+^-dependent inactivation [28,29].

The whole-cell compartmental model employed the Ultra-fast Monte Carlo algorithm introduced in the (Williams, Chikando et al. 2011) for channel gating, with 20,000 CRUs per cell, and was simulated using Fermi CUDA-capable GPGPU [20,30]. The single cell model is incorporated into a tissue model in which the individual myocytes are coupled to the four neighboring myocytes using electrical coupling. The membrane voltage of each myocyte is governed by

(1) $$\frac{dV_{m}}{dt}=-\frac{1}{C_{sc}}\left[\begin{array}{c} \frac{\left(I_{dhpr}^{T}+I_{dhpr,nj}\right)}{A_{m}}+I_{Na}+I_{NCX}+I_{NaK}+I_{pmca}+I_{K1}+I_{Kss}+I_{Ktof}\\ +I_{Ktos}+I_{background}+I_{app}+\overset{n}{\underset{k=1}{\sum }}G_{gap}^{k}(V_{m}^{k}-V_{m}) \end{array}\right]$$

where *V_m_* is the transmembrane membrane potential, *C_sc_* is specific membrane capacitance. *I_Na_* is the whole cell Na^+^ current and the two extraction pathways for the calcium via SL (sarcolemmal) are the plasma-membrane Ca^2+^/ATPase (PMCA) and the Na^+^/Ca^2+^ exchanger (NCX). *I_background_* represents three background currents, Ca^2+^, Na^+^, and K^+^ that follow the linear Ohm’s Law. Aside from the background current and the Na^+^/K^+^ exchanger, there are four other K^+^ currents. The formulas for fast and slow transient outward currents (*K_tof_* and *K_tos_*, respectively) were created for mice by [31] for the detailed equations of each individual ionic currents, readers are advised to check our recently published articles [11,19]. The term n is the number of coupled neighbors and *G^k^_gap_* is the gap junction conductance between the myocytes and its kth neighbor. The tissue model has provided some insights into the number of myocytes, under different conditions, required to generate an ectopic heartbeat or sustained spontaneous AP propagation across the tissue.

### 2.2. Numerical Methods

The system of ordinary differential equations comprising the model is solved using the explicit Euler method. The simulation uses no-flux boundary conditions based upon the assumption that there was not a significant gradient across cells at the border of the simulation. The adaptive timestep (10–100 nanoseconds) is required for numerical stability and is also necessary to capture the fast and stochastic gating of DHPR (dihydropyridine receptor) and RyR2 channels. Each release site uses a different sequence of pseudo-random numbers to control channel gating. These Monte Carlo simulations are computed on Fermi-GPGPU cards, with pseudo-random numbers derived from the Saru pseudo-random number generator on GPGPU provided by Asfar et al. [32]. When the channel fires, a smaller time-step is selected; first to ensure numerical stability, second to limit maximum 10% of the CRUs having state changes to occur at a specified time [33,34]. Channel gating depends upon the local dyadic subspace (Ca^2+^). The myoplasmic calcium indirectly links all the release sites through modulation of the subspace (Ca^2+^). Thus, the fraction 10% was selected so that the amount of calcium release does not change much in the bulk myoplasm of the system so that these CRUs are assumed to experience the same bulk myoplasmic calcium.

## 3. Results

The modeling study considers two possible mechanisms for the initiation of any arrhythmia: (1) current injection to replicate the membrane channel activation of the action potential, and (2) spontaneous Ca^2+^ release to model a Ca^2+^-entrained arrhythmia.

### 3.1. Current Injection

**Figure 1:**
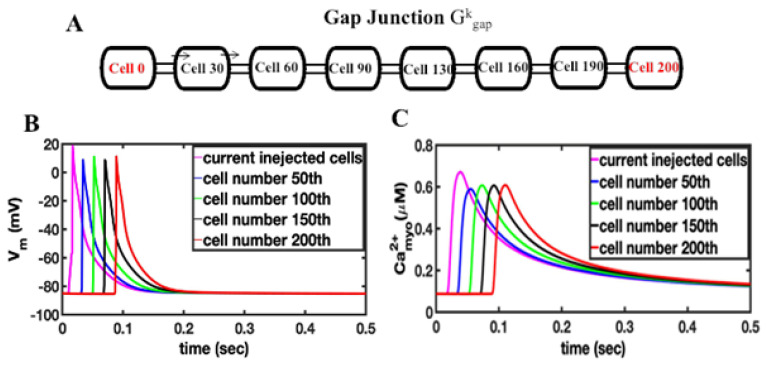
(**A**) Schematic of a 1-D tissue, myocytes are coupled by the gap junction conductance G^k^_gap_. Stimulus is applied to the end of the cable at cell 0 or cell 200. (**B**). Current injection into the first 7 myocytes (magenta) results in propagating action potential in 1-D cable of myocytes, (**C**) the current-injection induced action potential triggers calcium release in the 1-D cable of myocytes.

**Figure A1:**
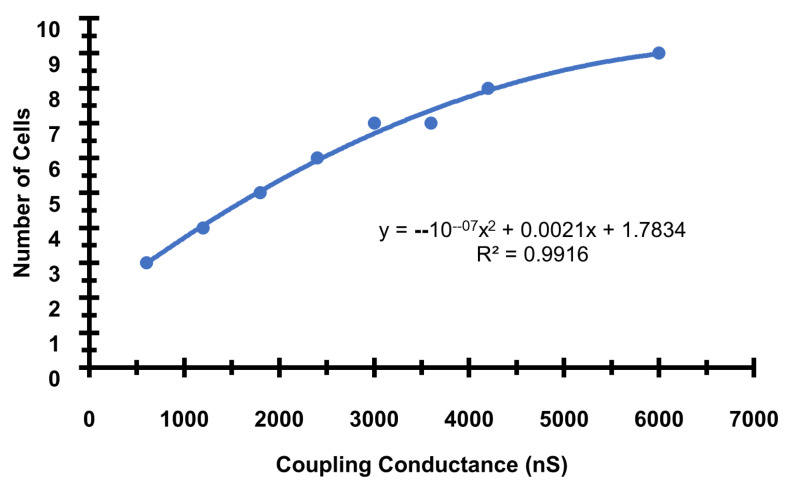
The number of cells needed to initiate a propagating AP increases as the gap junction conductance increases. The base gap junction conductance used in the simulations is 2400 ns. The formula and R^2^ for trendline is included in the inset.

**Figure A2:**
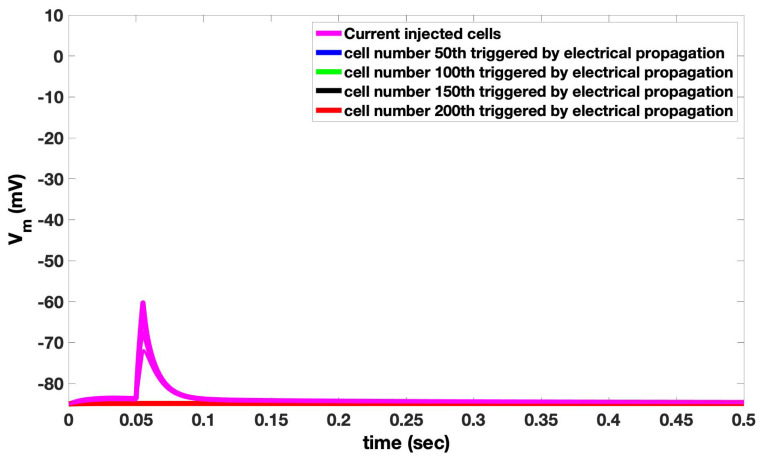
1-D Simulation with 5 cells fail to produce an action potential that can propagate between cells. Cell numbers 50, 100, 150, and 200 overlap.

The first set of simulations explores how the depolarization of cardiac ventricular myocytes by current injection can initiate an arrhythmia. The compartmental rat ventricular myocytes model developed previously is integrated into a network of myocytes to simulate one-dimension (1-D), two-dimension (2-D), and three-dimension (3-D) tissues [20]. Simulations in 1-D, 2-D, and 3-D represent physical structures in the heart (see [17] for more details). Simulations in 1-D can represent fiber strands such as Purkinje or cardiac trabeculae [18]. Simulations in 2-D represent myocardia sheets reflecting that the heart is arranged in fiber sheets with the conductivity in the sheet being greater than between sheets. The 3-D simulations can represent blocks of heart tissue such as the ventricular wall. The introduction of a “coarse grained model”, that describes a cardiac myocyte as a 1-D lattice of 200 independent Ca^2+^ release sites (each of these sites represents the behavior of 100 identical Ca^2+^ release sites to achieve a model with 20,000 total Ca^2+^ release sites), increases computational efficiency. Here the depolarization during an AP propagates from a cardiac myocyte to an adjacent no-refractory myocyte due to a fire–diffuse–fire mechanism. The 1-D chain of myocytes is stimulated by current injection that is applied at one end of the cable (Figure 1, red region), all myocytes in the cable are connected by gap junctions with conductance G^k^_gap_, i.e., gap junctions enable propagation of the cardiac action potential from cell to cell. Figure 1A shows the schematic of 1-D tissue. In order to generate a sustained propagating action potential in 1-D tissue, current needs to be injected into only seven myocytes (magenta), as shown in Figure 1B. In these simulations, the gap junction conductance is estimated at 2500 nS for the AP conductance velocity V_c_ to be 0.36 m/s, which is in the range of experimentally reported values [35,36]. The number of cells needed to initiate an arrhythmia will increase as the value of the gap junction conductance increases (Appendix B Figure A1). There is a close relationship between intracellular Ca^2+^ and the membrane potential in cardiac myocytes. When the cell depolarizes, Ca^2+^ influx through open L-type Ca^2+^ channels in the cell membrane triggers the release of Ca^2+^ by RyR2s on the sarcoplasmic reticulum into the dyad resulting in the myoplasmic Ca^2+^ transient. A simulated propagating action potential and the accompanying triggered Ca^2+^ wave is shown in Figure 1C. The excitation of seven myocytes is needed to initiate a propagating action potential. Appendix B Figure A2 shows that if five cells are activated, an action potential that can propagate to adjacent cells is not produced.

**Figure 2:**
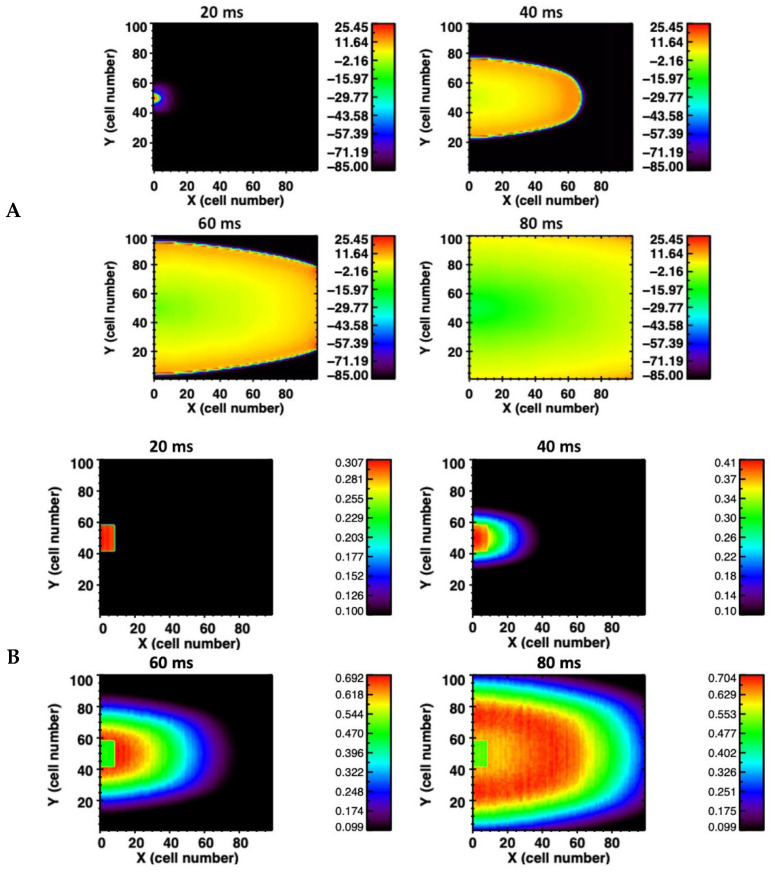
(**A**) Action potential propagation is shown at different times (20 milliseconds (ms), 40 ms, 60 ms, and 80 ms) during the simulation in the 2-D network of 100 × 100 rat ventricular myocytes. (**B**) The corresponding Ca^2+^ wave propagation in the 2-D network.

The simulations were extended into 2-D and 3-D tissue. In the 2-D homogeneous sheet, a 100 × 100 myocyte grid with anisotropic gap conductance is used. In this case the gap junction conductance is about three-fold (800 nS) greater along the longitudinal direction compared to the transverse direction. The anisotropy of conductance is visible in a 2-D tissue image in the propagation of AP shown in Figure 2A and the Ca^2+^ wave propagation in Figure 2B. Due to the anisotropy of gap junction conductance, propagation is faster (elliptical shape) along the *x*-axis compared to the *y*-axis. The propagation of AP and Ca^2+^ waves are shown in the Supplementary Videos S1 and S2. For the propagation of AP in 2-D tissue the number of myocytes increased to 49 myocytes and for the 3-D simulation (100 × 100 × 12 cells) with the anisotropy gap conductance the number of myocytes needed for the propagating AP was only 294. The 2-D simulations of AP and Ca^2+^ release propagation illustrated in Figure 2A used a network of 100 × 100 rat ventricular myocytes with stochastic Ca^2+^ dynamics. When the myocytes receiving the simulation are not adjacent (spatially random stimulation pattern) the number of myocytes needed increases significantly.

Simulation studies also investigated the response of the myocytes in tissue when an instantaneous and gradual current injection was applied by keeping the mass of the stimulus constant. In case of a gradual stimulus, a current of −6.52 μA/cm^2^ was injected for 5 ms for a total current mass of the stimulus to be −32.60 μA/cm^2^·mS. For the instantaneous case, a stimulus of −16.30 μA/cm^2^ was applied for only 2 ms to yield the same total stimulus current mass. In the instantaneous case, the number of myocytes to produce a sustained propagating AP was reduced. Since myocytes in the tissue are coupled by gap junction conductance G_gap_^k^, the gradual current injection dissipates to the neighboring myocytes making it hard for the simulated myocytes to depolarize. On the other hand, with a large duration current injection of a short duration there is sufficient current to depolarize in the tissue. The numbers of cells and percentage of cells needed for generating a successful propagation of depolarization when the current is injected instantaneously and gradually are given in Table 1. The numbers are comparable in 1-D to experimental observations in Purkinje fibers [37].

### 3.2. Spontaneous Ca^2+^ Release during Heart Failure (HF)

Spontaneous Ca^2+^ release is another mechanism for generating depolarization in cells by the activation of NCX. Previous studies have used the whole-cell model to examine the changes in local calcium release in HF [38]. Here, simulations are performed to suggest that the mechanism behind, and the number of myocytes needed to trigger a propagating AP in a tissue by implementing previously established changes in ion transport proteins: fast and slow K^+^ currents (I_to_ and I_K1_), were reduced by 20%; NCX protein expression was increased by 100%; SERCA2a protein was decreased by 30%; and RyR2 Ca^2+^ sensitivity was increased by 50% to mimic the increased activity from chronic hyperphosphorylation [7,39,40,41]. Our previous studies have reported the experimentally observed transverse tubule (TT) changes during HF [38]. In this study a computational model was developed to capture these changes. This included increased spacing between TTs and RyR2 clusters at CRUs of only 25% of TT junctions (whereas 75% of CRUs nanodomains remained unchanged), consistent with earlier studies [42]. This orphaning of RyR2 clusters during HF due to T-tubule remodeling was implemented for 25% CRUs through 30-fold increases in the subspace volume, whereas 75% of CRUs remains unchanged. The increase in subspace volume emulates the uncoupling between the L-type Ca^2+^ channel and RyR2 during an AP. The parameter values for normal and HF myocytes are described in the Appendix A.

**Figure 3:**
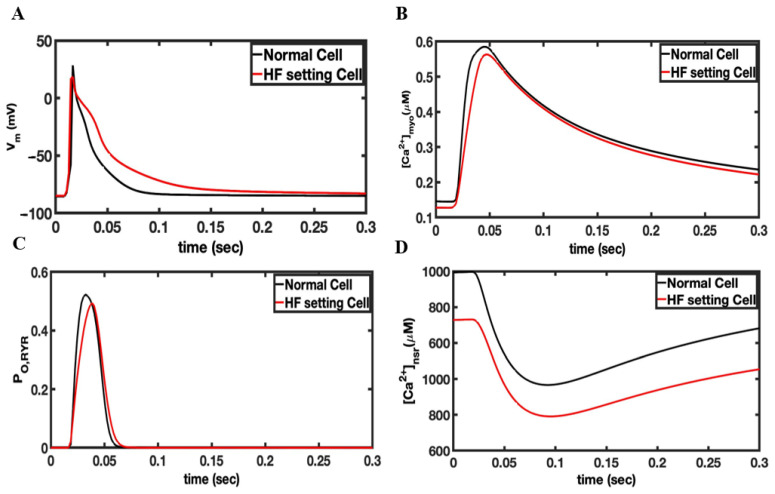
Comparison of simulated heart failure vs. control for the rat ventricular myocyte model. The model was paced in a single myocyte for 20 s so that steady-state pacing was obtained. (**A**) Changes in the membrane potential during an action potential. (**B**) The myoplasmic Ca^2+^ concentration during an AP. (**C**) The RyR2 open probability. (**D**) The network SR Ca^2+^ concentration.

In failing hearts, the increase in spontaneous Ca^2+^ leak is simulated by an increase in the opening rate constants. Figure 3 shows an AP generated by the rat ventricular myocytes under the control and the heart failure condition. The AP is slightly prolonged in simulated heart failure compared to the control (Figure 3A). The (Ca^2+^)_myo_ transient is slightly smaller and the diastolic level decreased during heart failure similar to the experimental observation in rat ventricular myocytes (Figure 3B) [43]. Increased RyR2 phosphorylation restores the RyR2 open probability in heart failure levels nearly similar to the control during the Ca^2+^ transient generated by the AP (Figure 3C). The diastolic RyR2 open probability is increased from 0.00051 for the control to 0.000204 for heart failure. The SR Ca^2+^ content is decreased in heart failure similar to the experiment (Figure 3D) due to the decreased SERCA expression and the increased diastolic Ca^2+^ leak. Note that this increased RyR2 open probability during heart failure helps to restore the (Ca^2+^)_myo_ transient to near-control levels. The delayed subcellular Ca^2+^ release and AP prolongation showed in the simulation results confirmed that the RyR2 cluster reorganization may contribute to dysfunctional AP and Ca^2+^ dynamics (Supplementary Videos S3 and S4). Ca^2+^ release is observed in cardiac myocytes by random opening of RyR2 channels located on the SR membrane. There is a possibility that Ca^2+^ waves can be triggered via the Ca^2+^-induced Ca^2+^ release phenomenon (CICR). CICR is the positive feedback property of the RyR2 that occurs when a rise calcium activates the RyR2 to release additional Ca^2+^. The rise in calcium can be from an influx from an L-type calcium channel, from spontaneous opening of the RyR2, or from the release of caged Ca^2+^. The abnormal rise of Ca^2+^ induced by these mechanisms will be refered to as a spontaneous Ca^2+^ wave. The rise of Ca^2+^ in the myoplasm caused by the Ca^2+^ wave activates the inward NCX current (forward mode I_NCX_), which triggers an extrasystole and hence depolarizes the myocytes to produce the sustained propagating AP in the myocardial tissue.

**Figure 4:**
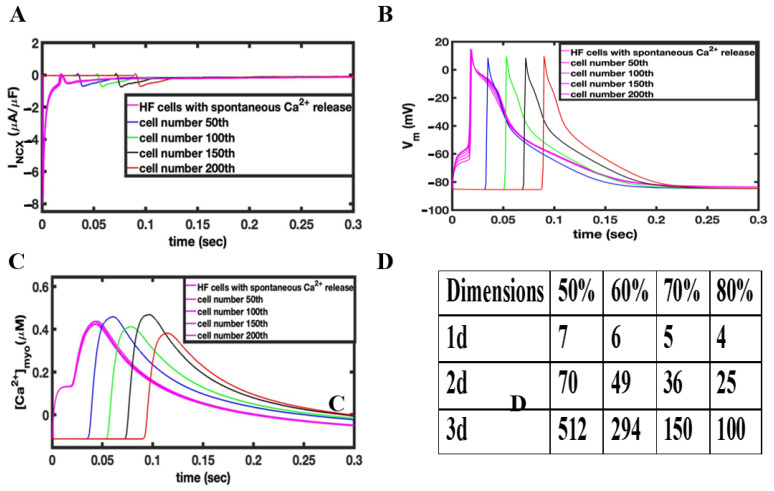
(**A**) Changes in I_NCX_ during the depolarization of myocytes due to the activating Ca^2+^ release. (**B**) Under HF conditions, opening 10 RyR2 channels in 50% of the release units results in a propagating action potential. (**C**) The Ca^2+^ rises slowly in the triggered myocytes resulting in depolarization. The action potential spreads rapidly to other myocytes activating calcium release. (**D**) Table showing dependence of number of myocytes to propagate AP as a function of the fraction of activated Ca^2+^ release units in 1-D, 2-D, and 3-D tissue.

In the simulations shown in Figure 4, 10 channels in a RyR2 cluster of about 49 RyR2s are opened to release Ca^2+^ into the dyadic cleft in 50% of the Ca^2+^ release units in HF phenotype myocytes. The intial Ca^2+^ release from the 10 activated channels in these HF myocytes will activate Ca^2+^ release in the remaining channels within the same RyR cluster. Figure 4A shows the NCX current in the tissue containing the failing myocytes (magenta). With the opening of the 50% of release a large forward mode NCX current is created because 20% of the NCX are in the dyadic space and thereore experience very high Ca^2+^ levels. This large NCX current facilitates ectopic depolarization caused by spontaneous Ca^2+^ release for failing myocytes. The AP produced in these failing myocytes propagates to the normal myocytes by the gap junction conductance G_gap_ as shown in Figure 4B. Only seven myocytes (magenta Figure 4B) were needed in 1-D to produce a propagating AP. Simulated 2-D and 3-D tissues implemented the same technique used in 1-D to generate propagating APs. The number of myocytes needed to depolarize the 2-D tissue with the sponatneous Ca^2+^ release increased from 7 in 1-D to 70 in 2-D and 512 in 3-D tissue. Table 2 shows the number of cell needed to trigger an AP by current injection and spontaneous Ca^2+^ release. Increasing the fraction of Ca^2+^ release units from 50% to 80% in simulated 1-D, 2-D, and 3-D tissues with the HF phenotype decreases the number of myocytes required to depolarize the tissue from 512 to 100 in 3-D tissue, from 70 to 25 myocytes in 2-D tissue, and from 7 to 4 in 1-D tissue, as shown in Figure 4D.

### 3.3. Influence of Cardiac Geometry

**Figure 5:**
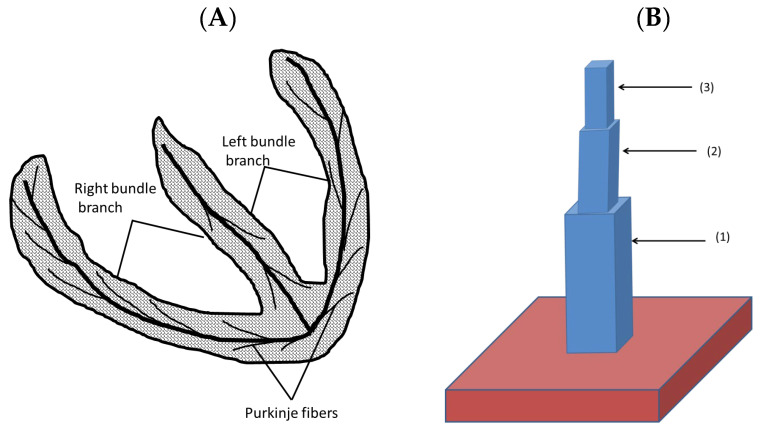
(**A**) The 1-D structure that extends from the atrioventricular bundle. (**B**) The computational approach representing a trabecula attached to the ventricular wall.

The heart has many fine structures such as Purkinje fibers and trabeculae (lining the chambers of the heart) that are in effect a 1-D structure as shown in Figure 5A. Studies have suggested that an arrhythmia originates in trabeculae or Purkinje fibers [37]. The simulations of 3-D blocks of tissues such as the ventricular tissues require hundreds of myocytes, which is potentially too large a number to generate an arrhythmia as all these myocytes would have to simultaneously depolarize. Cardiac trabeculae that are found on the inner wall of the ventricular lumen were simulated as 1-D structures connected to a 3-D mass simulating the heart wall. Figure 5B shows a schematic diagram of this structure with three rectangles (1), (2), and (3) of dimensions 6 × 6 × 7 myocytes, 4 × 4 × 6 myocytes, and 2 × 2 × 5 myocytes, respectively, on the top of a cube of 20 × 20 × 20 myocytes. Supplementary Videos S5 and S6 show the propagation in a normal and a failing heart, respectively.

**Figure 6:**
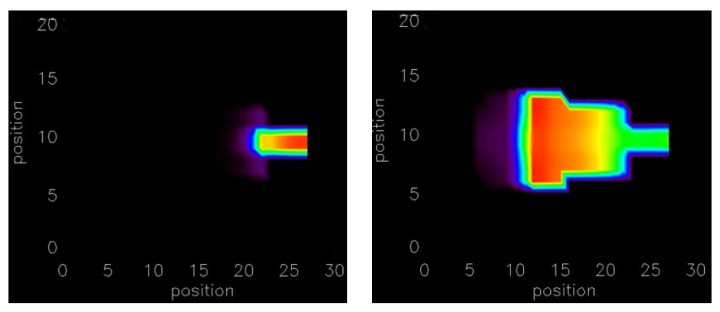

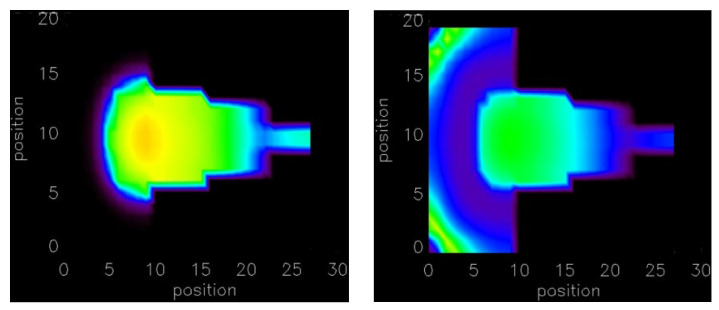
By injecting current results in a propagating action potential at along the line of 2 × 2 × 5 myocytes. The movies of the simulation are available in Supplementary Videos S5 and S6.

The depolarization of this 3-D structure by current injection and spontaneous Ca^2+^ release was simulated. Figure 6 shows the triggered AP at different times after injecting the current at the top of the trabecula as it propagates into the 3-D tissue. The gradually injected current at the top in 2 × 2 × 8 myocytes show that a total of 32 myocytes is sufficient to depolarize the myocytes to generate an AP propagating into the 3-D tissue (ventricular wall). The number of myocytes required for the case with instantaneous current injection is reduced to only 2 × 2 × 5 = 20 myocytes. The simulations suggest that initiation site geometry plays an important role in triggering AP. The 20–32 myocytes required for triggering arrhythmia by gradual current injection compared to 292 myocytes in the ventricular wall suggests that structures such as the trabeculae or Purkinje system are likely sites for the initiation of arrhythmia. In the case of failing heart tissue, the activation of 20 myocytes by gradual current injection in the trabecula is sufficient to initiate depolarization in the ventricular wall. If current is injected instantaneously then the number of myocytes is reduced to 20 from 100. In case of the spontaneous Ca^2+^ release in a failing heart, the number of myocytes is reduced to only 32 in the 1-D trabecula compared to 512 in the 3-D ventricular wall. The number is significantly smaller because the small size of the trabecular tissues reduced the electrotonic load that must be overcome to activate adjacent tissue.

### 3.4. Distributed Activation

**Figure 7:**
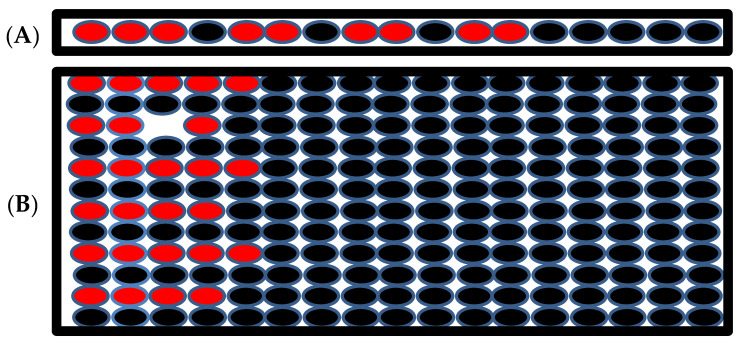
(**A**) The schematic of 1-D tissue where the current is injected into the myocytes with red filling, the myocytes with black filling are the unstimulated myocytes. (**B**). Schematic of the 2-D tissue when the stimulated myocytes are not neighbors but there are some unstimulated myocytes between them.

The final set of simulations tested the effect of current injection in a non-contiguous mass of myocytes rather than an adjacent cell mass. The first current was injected into alternate myocytes. In this simulation successful depolarization of these myocytes was not possible even if current was injected into many myocytes because of the continuous leak to the unstimulated myocytes. If a non-contiguous mass of cell as shown in Figure 7A is stimulated (red myocytes stimulated), 80% more myocytes (nine myocytes compared to five) are needed to produce depolarization in the 2-D tissue. The numbers of myocytes not only increased, but also needed to have placed at least three myocytes at the end of the cable for the depolarization of the myocytes.

In 2-D tissue the number of myocytes increased almost three-fold to produce successful depolarization of the tissue (Figure 7B). The red represents the stimulated myocytes, and the black represents the unstimulated myocytes in the tissue in the simulation. This simulation suggests that a large number of myocytes is needed to depolarize the tissue if the stimulated myocytes are not in a contiguous cell mass and suggests that even if a large number of uncoupled myocytes display an aberrant behavior, arrhythmia is unlikely the stability of normal heart rhythm. In order to generate arrhythmia, many myocytes in the close proximity need to display coordinated arrhythmogenic behavior.

## 4. Discussion

Cardiac arrhythmias require the aberrant behavior of a sufficient number of myocytes so that they can disrupt the normal depolarization pattern of the heart. This is true whether the arrhythmia is initiated by an ectopic focus or by the excitability/action potential dynamics of the cardiac myocyte. When an ectopic focus elicits a propagating action potential in front of the normal propagating depolarization wave front it can break the wave front, which can lead to spiral waves that are responsible for ventricular tachycardia, polymorphic ventricular tachycardia, and fibrillation [44]. In order for an ectopic focus to exist, it has to be protected from resetting by the normal depolarizing wave, which can occur due to a non-conductive zone (ischemia, fibrosis, and necrosis) or have a spontaneous release triggering a depolarization after the normal wave has passed [44,45,46,47]. Earlier modeling studies have suggested that a large number of myocytes (>700,000 myocytes) were required to initiate an arrhythmia [14]. Experimental studies that motivated their work indicated that an implanted mass of myocytes in the heart wall to take over heart pacing was approximately this size [15]. For such an implanted structure, the connections to the rest of the heart wall were most likely less well developed than connections between myocytes in the heart wall. Other simulation studies indicated that for the sinoatrial (SA) node, the depolarization of 1000 myocytes were needed to serve as an ectopic focus to initiate an arrhythmia [48]. Furthermore, the fine strands of atrial tissue that penetrate into and interdigitate with the SA node help to overcome the electrotonic loading effects [49].

The contribution of the Na^+^–Ca^2+^ exchange to Ca^2+^ removal varies across species. In rat ventricular myocytes, 92% of the Ca^2+^ transient is removed by SERCA2a and 7% by the NCX. In humans and rabbits, 70–75% of the Ca^2+^ transient is removed by SERCA2a, respectively, and 20–25% by the NCX [50,51]. Ca^2+^ sparks in paced rat and human ventricular myocytes are similar. Ca^2+^ sparks in human ventricular myocytes have been observed to have a F/F0 of 1.3 in normal heart and 1.19 in a failing heart [52]. Ca^2+^ sparks in rat ventricular myocytes have been reported to have a F/F0 of 1.2–1.6 in paced ventricular myocytes [53,54]. However, in resting rat ventricular myocytes the F/F0 has been measured as high as 3.5–4 in the control and 3.0 post-myocardial infarction ventricular myocytes due to loading of the SR. These two observations suggest that there would be more depolarizing current through Na^+^–Ca^2+^ exchange in human and rabbit ventricular myocytes and hence be easier to generate an arrhythmia.

**Figure A3:**
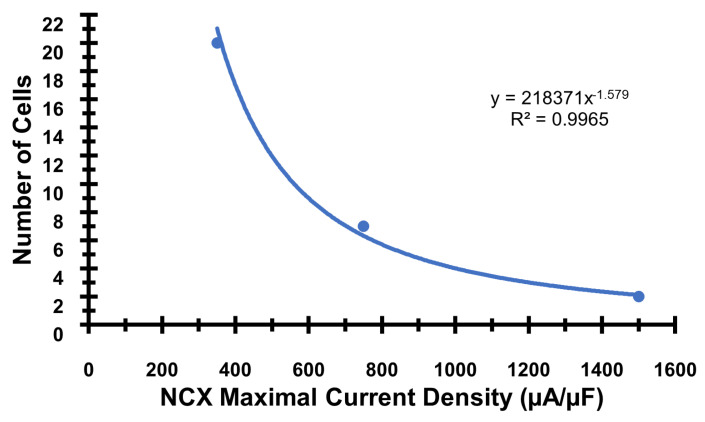
The number of cells needed to initiate a propagating AP increases as the maximal Na^+^-Ca^2+^ exchanger maximal current density increases (equivalent to increase expression. The base value of v_ncx_ used in the simulation is 750 s^−1^. The formula and R^2^ for trendline is included in the inset.

The changes to the NCX expression during heart failure varies between species [55]. In humans, some studies have failed to find a correlation between NCX expression levels and the different stages of heart failure [56]. Simulations performed at different levels of expression indicated that with an increase NCX expression, there is a greater likelihood of generating an action potential that can propagate. This is consistent with the experimental findings that in heterozygous NCX knock-out mouse ventricular myocytes, the occurrence of delayed afterdepolarizations and early afterdepolarizations is reduced compared to the wild type [57]. However, it should be noted that if the aberrant Ca^2+^ release is larger, a smaller amount of the NCX overexpression is needed to generate a propagating arrhythmia. Appendix B Figure A3 shows how the number of cells needed to initiate a propagating AP decreases with increasing the NCX maximum current density. Studies in humans have observed that NCX protein levels were inversely correlated with the frequency-dependent rise in the diastolic force [39]. Interestingly, the NCX protein levels have been positively correlated with the plasma norepinephrine levels in heart transplant patients [56].

Similarly, the changes to Ca^2+^ dynamics will also vary across species and with the extent of progression of the heart failure. In compensated heart failure, beta adrenergic stimulation is able to rescue the myoplasmic Ca^2+^ transient to be close to normal. In uncompensated heart failure the Ca^2+^ transient is decreased in amplitude and of a prolonged duration. In the case of decompensated heart failure, the AP can also be very prolonged. In the model presented here, the Ca^2+^ transient amplitude is only slightly decreased from the control because the beta-adrenergic stimulation has increased the RyR opening. The diastolic myoplasmic Ca^2+^ level can also vary being elevated, decrease, or unchanged depending upon the species, pacing rate, and extent of heart failure in human patients even with depleted SR Ca^2+^ [58]. The model suggests that this is likely related to the changes in NCX expression since the NCX controls the total intracellular Ca^2+^. In cases where there is upregulation of the NCX the diastolic myoplasmic Ca^2+^ will be lower [58,59].

Previous studies have shown that changes to the T-tubular network also influence the propensity for spontaneous Ca^2+^ release and waves [60,61]. Factors such as the increased distance between sites can limit Ca^2+^ wave propagation and the increased amount of RyR2 phosphorylation can enhance wave propagation. Our earlier studies have reported the experimentally observed transverse tubule changes during HF [38]. In this study a computational model was developed to capture these changes. This included increased spacing between TTs and RyR2 clusters at CRUs of only 25% of TT junctions (whereas 75% of the CRUs nanodomains remained unchanged), consistent with earlier studies [42]. This orphaning of RyR2 clusters during HF due to T-tubule remodeling was implemented for 25% of CRUs through 30-fold increases in the subspace volume, whereas 75% of the CRUs remain unchanged. The increase in subspace volume emulates the uncoupling between L-type Ca^2+^ calcium channel and RyR_2_ during an AP.

The model simulated the major changes associated with heart failure. There are additional changes that could be studied in the future. For example, the scientific literature has reports that phospholamban changes during heart failure and other reports where it does not [62]. A more recent study claims that there is little evidence of phospholamban down-regulation in heart failure [63]. Due to the controversy over these changes, we have not included the effect of changes in phospholamban expression.

A simulation series was performed to understand the importance of synchrony of excitation in the excited cell mass. When the release in different myocytes in a cluster are temporally distributed, that is, the triggering Ca^2+^ release events do not happen synchronously, it becomes more difficult to generate a propagating depolarization as the temporal spread of myocyte depolarizations gets larger. However, as long as the depolarizations overlap such as would occur if the depolarization spread from cell to cell, a small number of cells is still needed. Further studies are needed to explore how a Ca^2+^ wave can spread between cells to form the mass of cells needed to trigger an arrhythmia.

The simulations presented here indicate that in 1-D-like structures, the excitation of as little as four myocytes is required to initiate a propagating action potential to the rest of the structure in agreement with experimental findings [37]. In fact, if the arrhythmia is initiated in a1-D-like structure such as a cardiac trabecula, Purkinje fiber, or the atrial penetrations into the SA node, the number of myocytes to propagate excitement to a mass of tissue such as the ventricular wall is small, as little as 20 myocytes. This can explain the experimental finding that arrhythmias originate in the trabeculae or Purkinje fibers. It can also explain how in damaged/fibrosed tissue the non-conductive scar tissue can isolate lower dimensional structures that lower the threshold for initiating an arrhythmia. The simulations suggest that in a 2-D sheet of myocytes. As few as 36 cells are needed for an ectopic focus to initiate a cardiac arrhythmia. In experiments with a cultured 2-D sheet of ventricular myocytes, they found that 8-50 myocytes were sufficient to initiate an arrhythmia at the edge of an ischemic region consistent with simulations of a similar condition [46,47].

The ventricular wall has different cell types in the epicardium, midmyocardium, and endocardium. There is also a transmural gradient moving across the wall transmurally and from apex to base. In the 3-D simulations of a tissue, there was no transmural gradient included in the simulation because in the block of ventricular tissue the number of cells in the simulations were small compared to the wall thickness. For simplicity a single ventricular cell type was used. This is justified because during the activation of the action potential they all have a fast upstroke velocity varying by less than 10% [64]. Furthermore, studies have suggested that a difference in the action potential duration rather than the initiation are important for the generation of arrhythmia [65,66].

In this study, compartmental models of the rat ventricular myocyte were used instead of a spatially resolved model. The simulation of spatially resolved ventricular myocytes can simulate spontaneous Ca^2+^ waves and the mechanisms that contribute to their initiation and propagation [19]. Experimental studies have shown that a spontaneous Ca^2+^ wave can be accompanied by the depolarization of the myocyte [67]. The depolarization will activate more Ca^2+^ release units. Hence, activation of 50% of the release units was used to simulate spontaneous Ca^2+^ release in the ventricular myocytes. Because depolarization of a single ventricular myocyte is uniform across the cell, compartmental models of the ventricular myocyte are sufficient for this study. The computationally expensive stochastic local control model used has advantages over the computationally fast common pool model. The local control model produces graded release and captures the important interactions between SR Ca^2+^ release and the Ca^2+^-dependent inactivation of the L-type channel, which the common pool model fails to accomplish [11,20,38,68].

The origination of ectopic foci in both atrial and ventricular tissue has been studied previously. Atrial arrhythmia can originate at the pulmonary veins [69]. Other veinous structures have also been found to serve as the initiation sites for arrhythmias [70]. The simulations shown here demonstrate how a reduction in the dimensionality of the system can lower the number of myocytes needed for the generation of an arrhythmia. Structures such as the pulmonary veins effectively reduce the dimensionality from 3-D to 2-D. Furthermore, the ventricular wall structure contains sheets of cells that also could have a reduced dimensionality. The Purkinje fiber system and trabeculae have been identified as a source for arrhythmia [71,72,73,74,75]. These arrhythmias can arise as a result of ion channel dysfunction, for example through mutation. Alternately, aberrant Ca^2+^ dynamics can also be a cause of these arrhythmia [71]. The Purkinje system and trabeculae are effectively a 1-D structure that serves as the conduction system to excite the ventricular tissue. In the Purkinje system, experiments show that as little as four myocytes need to depolarize together to create a propagation depolarization [37]. The simulations shown in the current study agree with this experimental finding that only a small number of myocytes are needed to trigger an arrhythmia given the appropriate geometry. Other studies have shown that arrhythmia can initiate in the trabeculae [18,75]. The trabeculae are columnar strands of muscle that are found on the interior of the ventricular walls. They effectively form a 1-D structure from which an arrhythmia can initiate with a small number of myocytes similar as demonstrated in the model simulations. Arrhythmia occurring during ischemia and reperfusion have been shown to involve calcium overload, particularly in the border zone between the ischemic and perfused regions [47,76]. In this border zone, gap junctions have been observed to decrease by 40% [77]. The combination of these two factors results in an increase in spontaneous depolarization and a reduction in dimensionality that can increase the possibility of the initiation of an arrhythmia by a mechanism described by the simulations in this paper.

Experimental studies have considered the remuscularization of the heart as a means of repairing damaged heart muscle [78,79]. Multiscale modeling studies have demonstrated that for implanted tissues, arrhythmias tend to initiate at the border of the implanted tissue [80]. This implanted tissue has been suggested to have a lower oxygenation that can impact graft survival [81]. This can lead to an ischemic region that can serve as an initiation site for arrhythmia, as described above.

One important question not addressed by the current modeling study is how a mass of myocytes synchronize to serve at the ectopic focus for initiating an arrhythmia. This has been studied in other modeling studies. Previous modeling studies have considered the coupling of an SA node cell using a 7 × 7 grid of myocytes [82]. These studies demonstrated how the coupling between myocytes plays a role in the synchronous firing of the SA node. A more recent modeling study demonstrated using a simplified model that at low pacing frequencies Ca^2+^ waves do not contribute to synchronization [83]. However, at faster pacing frequencies the Ca^2+^ wave can synchronize over millions of myocytes and lead to dramatic action potential fluctuations. The modeling in this paper does not spatially resolve individual myocytes and instead treats the myoplasm as one compartment as described previously [11]. The contribution of Ca^2+^ waves can be studied using our stochastic spatial rat ventricular myocyte model and is left for future work [19].

The model predictions of the critical number of cells needed to initiate an ectopic propagating action potential suggest that a small number of cells is needed. These results might vary with other computational models for rat or for other species. For example, Xie and co-workers found very different numbers using a different model with different assumptions about the system being modelled [84]. For example, Wilhelms and co-workers discovered differences when using alternate atrial myocyte models on action potential properties in 1-D, 2-D, and 3-D [85]. Furthermore, modeling studies in ventricular myocytes have shown that the Na^+^ channel dynamics can change model behaviors during arrhythmia [86,87]. This is a limitation of the current study. Future work can explore the effect of different models for rat and other species on the number of cells. Alternately, a perturbation analysis of the current model to explore how changes to the model affect simulation predictions similar to the approach used by Sutanto can be applied [88].

## 5. Conclusions

The experimentally constrained computational model developed here permits an understanding of how Ca^2+^ sparks trigger spontaneous Ca^2+^ release in the rat ventricular myocyte. The detailed 1-D, 2-D, and 3-D modeling of myocytes enables the investigation of how Ca^2+^ instability arises, and how it may lead to novel electrical activity and arrhythmia. The model presented here supports the idea that spontaneous calcium release during conditions such as heart failure or in an EAD can result in an action potential through the activation of NCX. This is a Ca^2+^-activated electrogenic transport protein and is one of the critical components of the heart cell that links (Ca^2+^)_i_ changes with membrane current. Thus, when there is a large increase in (Ca^2+^)_i_, as there is with every heartbeat, there is a parallel inward (depolarizing) current. Abnormal Ca^2+^ release events activate the NCX current (I_NCX_), which may trigger an extrasystole and hence produce an arrhythmia. The subcellular and nanoscopic characterization of (Ca^2+^)_i_, which activates I_NCX_, has also been investigated. This requires high temporal and spatial resolution modeling of local Ca^2+^ signals originating from the junctional sarcoplasmic reticulum (JSR). Both aspects of this behavior have been investigated.

Simulations suggest that in order to generate depolarization in the heart, the myocytes need to be in close proximity, and only then a small number of myocytes will be able generate arrhythmia in heart. If these stimulated myocytes were not close to each other, then number of the myocytes to produce successful depolarization increased a lot. We conclude that this could be the reason that makes arrhythmia a rare event. Finally, the simulations suggest that a relatively small number of myocytes are needed to trigger a propagating action potential with the number increasing as the dimensionality increases from 1-D to 2-D to 3-D. Fine structures in such as Purkinje fibers and trabeculae provide a virtual 1-D media in which an arrhythmia can be initiated. This greatly reduces the number or myocytes needed to initiate an arrhythmia in the 3-D heart.

## Figures and Tables

**Table 1:** Number and percentage of myocytes with the instantaneous and gradual current injection in 1-D, 2-D, and 3-D tissue.

Protocol	1-D	2-D	3-D
Gradual	7 (7%)	49 (0.49%)	294 (0.24%)
Instantaneous	5 (5%)	36 (0.36%)	100 (0.083%)

**Table 2:** The number of myocytes needed to trigger propagation of action potential.

Protocol	1-D	2-D	3-D
Current injection	7 (7%)	49 (0.49%)	294 (0.24%)
Calcium release during HF	7 (7%)	70 (0.70%)	512 (0.43%)

## Data Availability

Model code is complex and might need explanation. The codes will be made available upon request to aullah3@gmu.edu.

## Supplementary Materials

The following supporting information can be downloaded at: https://www.mdpi.com/article/10.3390/cells11121878/s1, Video S1: Simulated action potential propagation across normal ventricular myocytes in a 100 × 100 network initiated by current injection; Video S2: Simulated action potential triggered calcium release across normal ventricular myocytes in a 100 × 100 network initiated by current injection; Video S3: Simulated action potential propagation across failing ventricular myocytes in a 100 × 100 network initiated by current injection; Video S4: Simulated action potential triggered calcium release across failing ventricular myocytes in a 100 × 100 network initiated by current injection; Video S5: Simulated action potential propagation across normal ventricular myocytes in a simulated trabeculae connected to the ventricular wall initiated by current injection; Video S6: Simulated action potential propagation across failing ventricular myocytes in a simulated trabeculae connected to the ventricular wall initiated by current injection. Model Parameters are available in Appendix A below. The rat ventricular myocyte model was recently published, and the parameters described below are for the normal and HF setting. Please check [11,19] for the detail and description of the parameters of the model and the Data Availability Statement.

## Appendix A

**Table A1:** Constant data and whole-cell level data.

Variables	Value	HF Setting	Unit
Faraday constant (F)	9.6485 × 10^4^		C/mol
Universal gas constant (R)	8.314 × 10^3^		mJ/(mol·K)
Temperature (T)	310		K
[Na^+^]_o_	1.4d5		µM
[Ca^2+^]_o_	1.8d3		µM
[K^+^]_o_	5.4 × 10^3^		µM
Cell volume (V_cell_)	25.0		pL
Myoplasmic volume (V_myo_)	12.5		pL
Network SR volume ($V_{nsr}^{}$)	0.762		pL
Junctional SR volume ($V_{jsr}^{T}$)	1.25 × 10^−1^		pL
Subspace volume ($V_{ds}^{T}$)	1.55 × 10^−2^		pL
Concentration [SERCA2a] K_d,myo_ K_d,sr_	300 900 2150		µM µM µM
RyR2 release rate ($v_{ryr}^{T}$)	56.4279		1/s
Percent nj-RyR2	0.5		Unitless
Transfer rate from subspace to bulk myoplasm ($v_{efflux}^{T}$)	300		1/s
Refill rate from nSR to jSR ($v_{refill}^{T}$)	2.4		1/s
NCX maximum current density ($\bar{I_{ncx}}$) K_d,ncxNa_ K_d,ncxCa_	520 8750 1380	1040	µA/µF
PMCA maximum current density ($\bar{I_{pmca}}$) K_d,pmca_	0.12 0.5		µA/µF
Na^+^/K^+^ maximum current density ($\bar{I_{Na/K}}$)	0.88		µA/µF
SERCA2a Pump Concetration Ap	300	240	µM
g_K1_ g_Kss_ g_Ktof_ g_Ktos_	0.1 0.00421 0.0798 0.00629	0.07 0.001263 0.05586 0.004403	mS/µF
Csc	1	0.8	µA/cm^2^
RyR2_2_ Ca^2+^ sensitivity	12	18	Unitless

**Table A2:** Initial values.

Variables	Value	Unit
m_Na_	0.0015	Unitless
h_Na_	0.9849	Unitless
j_Na_	0.9849	Unitless
a_Kss_	0.0021	Unitless
in_Kss_	1	Unitless
a_Ktof_	0.0021	Unitless
in_Ktof_	1	Unitless
a_Ktos_	2.9871 × 10^−4^	Unitless
in_Ktos_	0.9994	Unitless
[Ca^2+^]_myo_	0.08769	µM
[Ca^2+^]_nsr_	1.00737 × 10^3^	µM
[Na^+^]_i_	1.02 × 10^4^	µM
[K^+^]_i_	1.4372 × 10^5^	µM

**Table A3:** Calcium buffers.

Variables	Value	Unit
Myoplasmic buffer concentration K_d_	123 0.96	µM µM
jSR buffer concentration (e.g., Calsequestrin) K_d_	1.4 × 10^4^ 638	µM µM
High-affinity Troponin C concentration $k_{CM}^{+}$ $k_{CM}^{-}$	140 2.37 3.2 × 10^−2^	µM 1/(µM·s) 1/s
Calmodulin concentration $k_{SR}^{+}$ $k_{SR}^{-}$	24 30 71.4	µM 1/(µM·s) 1/s
SL buffer concentration $k_{SL}^{+}$ $k_{L}^{-}$	250 115 1000	µM 1/(µM·s) 1/s
SR buffer concentration $k_{SR}^{+}$ $k_{SR}^{-}$	47 115 100	µM 1/(µM·s) 1/s

## References

[1] Shiferaw Y., Aistrup G.L., Wasserstrom J.A. (2012). Intracellular Ca^2+^ Waves, Afterdepolarizations, and Triggered Arrhythmias.

[2] Janse M.J. (2004). Electrophysiological changes in heart failure and their relationship to arrhythmogenesis. Cardiovasc. Res..

[3] van Gorp P.R., Trines S.A., Pijnappels D.A., de Vries A.A. (2020). Multicellular in vitro models of cardiac arrhythmias: Focus on atrial fibrillation. Front. Cardiovasc. Med..

[4] Szabó Z., Ujvárosy D., Ötvös T., Sebestyén V., Nánási P.P. (2020). Handling of Ventricular Fibrillation in the Emergency Setting. Front. Pharmacol..

[5] Srinivasan N.T., Schilling R.J. (2018). Sudden Cardiac Death and Arrhythmias. Arrhythm. Electrophysiol. Rev..

[6] Denham N.C., Pearman C.M., Caldwell J.L., Madders G.W.P., Eisner D.A., Trafford A.W., Dibb K.M. (2018). Calcium in the Pathophysiology of Atrial Fibrillation and Heart Failure. Front. Physiol..

[7] Lehnart S.E., Wehrens X.H., Reiken S., Warrier S., Belevych A.E., Harvey R.D., Richter W., Jin S.-L.C., Conti M., Marks A.R. (2005). Phosphodiesterase 4D deficiency in the ryanodine-receptor complex promotes heart failure and arrhythmias. Cells.

[8] Chudin E., Goldhaber J., Garfinkel A., Weiss J., Kogan B. (1999). Intracellular Ca^2+^ dynamics and the stability of ventricular tachycardia. Biophys. J..

[9] Kistamás K., Veress R., Horváth B., Bányász T., Nánási P.P., Eisner D.A. (2020). Calcium Handling Defects and Cardiac Arrhythmia Syndromes. Front. Pharmacol..

[10] Sutanto H., Lyon A., Lumens J., Schotten U., Dobrev D., Heijman J. (2020). Cardiomyocyte calcium handling in health and disease: Insights from in vitro and in silico studies. Prog. Biophys. Mol. Biol..

[11] Hoang-Trong M.T., Ullah A., Lederer W.J., Jafri M.S. (2021). Cardiac Alternans Occurs through the Synergy of Voltage-and Calcium-Dependent Mechanisms. Membranes.

[12] Pott C., Eckardt L., Goldhaber J.I. (2011). Triple threat: The Na^+^/Ca^2+^ exchanger in the pathophysiology of cardiac arrhythmia, ischemia and heart failure. Curr. Drug Targets.

[13] Landstrom A.P., Dobrev D., Wehrens X.H.T. (2017). Calcium Signaling and Cardiac Arrhythmias. Circ. Res..

[14] Xie Y., Sato D., Garfinkel A., Qu Z., Weiss J.N. (2010). So little source, so much sink: Requirements for afterdepolarizations to propagate in tissue. Biophys. J..

[15] Plotnikov A.N., Shlapakova I., Szabolcs M.J., Danilo P., Lorell B.H., Potapova I.A., Lu Z., Rosen A.B., Mathias R.T., Brink P.R. (2007). Xenografted adult human mesenchymal stem cells provide a platform for sustained biological pacemaker function in canine heart. Circulation.

[16] Grace A.A., Roden D.M. (2012). Systems biology and cardiac arrhythmias. Lancet.

[17] Sutanto H., Heijman J. (2022). Integrative Computational Modeling of Cardiomyocyte Calcium Handling and Cardiac Arrhythmias: Current Status and Future Challenges. Cells.

[18] Vermeulen J.T., McGuire M.A., Opthof T., Coronel R., de Bakker J.M., Klöpping C., Janse M.J. (1994). Triggered activity and automaticity in ventricular trabeculae of failing human and rabbit hearts. Cardiovasc. Res..

[19] Hoang-Trong T.M., Ullah A., Lederer W.J., Jafri M.S. (2021). A Stochastic Spatiotemporal Model of Rat Ventricular Myocyte Calcium Dynamics Demonstrated Necessary Features for Calcium Wave Propagation. Membranes.

[20] Williams G.S., Chikando A.C., Tuan H.-T.M., Sobie E.A., Lederer W., Jafri M.S. (2011). Dynamics of calcium sparks and calcium leak in the heart. Biophys. J..

[21] Ullah A., Hoang-Trong T., Williams G., Lederer J., Jafri M. (2014). A small number of cells is sufficient to trigger a cardiac arrhythmia: Stochastic computational studies. Biophys. J..

[22] Tran K., Smith N.P., Loiselle D.S., Crampin E.J. (2009). A thermodynamic model of the cardiac sarcoplasmic/endoplasmic Ca^2+^ (SERCA) pump. Biophys. J..

[23] Sun L., Fan J.S., Clark J.W., Palade P.T. (2000). A model of the L-type Ca^2+^ channel in rat ventricular myocytes: Ion selectivity and inactivation mechanisms. J. Physiol..

[24] Groff J.R., Smith G.D. (2008). Ryanodine receptor allosteric coupling and the dynamics of calcium sparks. Biophys. J..

[25] Stern M.D., Song L.S., Cheng H., Sham J.S., Yang H.T., Boheler K.R., Rios E. (1999). Local control models of cardiac excitation-contraction coupling. A possible role for allosteric interactions between ryanodine receptors. J. Gen. Physiol..

[26] Sobie E.A., Dilly K.W., dos Santos Cruz J., Lederer W.J., Jafri M.S. (2002). Termination of Cardiac Ca^2+^ Sparks: An Investigative Mathematical Model of Calcium-Induced Calcium Release. Biophys. J..

[27] Liu O.Z., Lederer W.J., Sobie E.A. (2012). Does the Goldilocks Principle apply to calcium release restitution in heart cells?. J. Mol. Cell. Cardiol..

[28] Sobie E.A., Lederer W.J. (2012). Dynamic local changes in sarcoplasmic reticulum calcium: Physiological and pathophysiological roles. J. Mol. Cell. Cardiol..

[29] Terentyev D., Viatchenko-Karpinski S., Valdivia H.H., Escobar A.L., Gyorke S. (2002). Luminal Ca^2+^ controls termination and refractory behavior of Ca^2+^-induced Ca^2+^ release in cardiac myocytes. Circ. Res..

[30] Jafri M.S., Hoang-Trong T.M., Williams G.S.B. (2015). Method and System for Utilizing Markov Chain Monte Carlo Simulations. U.S. Patent.

[31] Bondarenko V.E., Szigeti G.P., Bett G.C., Kim S.-J., Rasmusson R.L. (2004). Computer model of action potential of mouse ventricular myocytes. Am. J. Physiol.-Heart Circ. Physiol..

[32] Afshar Y., Schmid F., Pishevar A., Worley S. (2013). Exploiting seeding of random number generators for efficient domain decomposition parallelization of dissipative particle dynamics. Comput. Phys. Commun..

[33] DeRemigio H., LaMar M.D., Kemper P., Smith G.D. (2008). Markov chain models of coupled calcium channels: Kronecker representations and iterative solution methods. Phys. Biol..

[34] Smith G.D., Fall C.P., Marland E.S., Wagner J.M., Tyson J.J. (2002). Modeling the Stochastic Gating of Ion Channels. Interdisciplinary Applied Mathematics. Computational Cell Biology.

[35] Rohr S. (2004). Role of gap junctions in the propagation of the cardiac action potential. Cardiovasc. Res..

[36] Rohr S., Kucera J.P., Kleber A.G. (1998). Slow conduction in cardiac tissue, I: Effects of a reduction of excitability versus a reduction of electrical coupling on microconduction. Circ. Res..

[37] Boyden P.A., Dun W., Robinson R.B. (2016). Cardiac Purkinje fibers and arrhythmias; The GK Moe Award Lecture 2015. Heart Rhythm.

[38] Wagner E., Lauterbach M.A., Kohl T., Westphal V., Williams G.S., Steinbrecher J.H., Streich J.H., Korff B., Tuan H.T., Hagen B. (2012). Stimulated emission depletion live-cell super-resolution imaging shows proliferative remodeling of T-tubule membrane structures after myocardial infarction. Circ. Res..

[39] Hasenfuss G., Schillinger W., Lehnart S.E., Preuss M., Pieske B., Maier L.S., Prestle J., Minami K., Just H. (1999). Relationship between Na^+^-Ca^2+^-exchanger protein levels and diastolic function of failing human myocardium. Circulation.

[40] Meyer M., Schillinger W., Pieske B., Holubarsch C., Heilmann C., Posival H., Kuwajima G., Mikoshiba K., Just H., Hasenfuss G. (1995). Alterations of sarcoplasmic reticulum proteins in failing human dilated cardiomyopathy. Circulation.

[41] Winslow R.L., Rice J., Jafri S., Marban E., O’Rourke B. (1999). Mechanisms of altered excitation-contraction coupling in canine tachycardia-induced heart failure, II: Model studies. Circ. Res..

[42] Song L.S., Sobie E.A., McCulle S., Lederer W.J., Balke C.W., Cheng H. (2006). Orphaned ryanodine receptors in the failing heart. Proc. Natl. Acad. Sci. USA.

[43] Gattoni S., Røe Å.T., Aronsen J.M., Sjaastad I., Louch W.E., Smith N.P., Niederer S.A. (2017). Compensatory and decompensatory alterations in cardiomyocyte Ca^2+^ dynamics in hearts with diastolic dysfunction following aortic banding. J. Physiol..

[44] Antzelevitch C., Burashnikov A. (2011). Overview of Basic Mechanisms of Cardiac Arrhythmia. Card. Electrophysiol. Clin..

[45] Alonso S., Dos Santos R.W., Bär M. (2016). Reentry and Ectopic Pacemakers Emerge in a Three-Dimensional Model for a Slab of Cardiac Tissue with Diffuse Microfibrosis near the Percolation Threshold. PLoS ONE.

[46] Wilders R., Wagner M.B., Golod D.A., Kumar R., Wang Y.-G., Goolsby W.N., Joyner R.W., Jongsma H.J. (2000). Effects of anisotropy on the development of cardiac arrhythmias associated with focal activity. Pflügers Archiv.

[47] Arutunyan A., Swift L.M., Sarvazyan N. (2002). Initiation and propagation of ectopic waves: Insights from an in vitro model of ischemia-reperfusion injury. Am. J. Physiol. Heart Circ. Physiol..

[48] Winslow R.L., Varghese A., Noble D., Adlakha C., Hoythya A. (1993). Generation and propagation of ectopic beats induced by spatially localized Na-K pump inhibition in atrial network models. Proc. Biol. Sci..

[49] Winslow R.L., Varghese A. Modeling the functional role of SA node-atrial interdigitation. Proceedings of the Computers in Cardiology 1994.

[50] Bers D.M. (2001). Book TitleExcitation-Contraction Coupling and Cardiac Contractile Force.

[51] Libby P., Zipes D.P., Bonow R.O., Mann D.L., Tomaselli G.F., Bhatt D. (2019). Braunwald’s Heart Disease: A Textbook of Cardiovascular Medicine.

[52] Lindner M., Brandt M.C., Sauer H., Hescheler J., Böhle T., Beuckelmann D.J. (2002). Calcium sparks in human ventricular cardiomyocytes from patients with terminal heart failure. Cell Calcium.

[53] Balnave C.D., Vaughan-Jones R.D. (2000). Effect of intracellular pH on spontaneous Ca^2+^ sparks in rat ventricular myocytes. J. Physiol..

[54] Cheng H., Lederer W.J. (2008). Calcium sparks. Physiol. Rev..

[55] Wang Y., Hill J.A. (2010). Electrophysiological remodeling in heart failure. J. Mol. Cell Cardiol..

[56] Schillinger W., Fiolet J.W., Schlotthauer K., Hasenfuss G. (2003). Relevance of Na^+^–Ca^2+^ exchange in heart failure. Cardiovasc. Res..

[57] Bögeholz N., Pauls P., Bauer B.K., Schulte J.S., Dechering D.G., Frommeyer G., Kirchhefer U., Goldhaber J.I., Müller F.U., Eckardt L. (2015). Suppression of Early and Late Afterdepolarizations by Heterozygous Knockout of the Na^+^/Ca^2+^ Exchanger in a Murine Model. Circ. Arrhythmia Electrophysiol..

[58] Eisner D.A., Caldwell J.L., Trafford A.W., Hutchings D.C. (2020). The Control of Diastolic Calcium in the Heart. Circ. Res..

[59] Asp M.L., Martindale J.J., Heinis F.I., Wang W., Metzger J.M. (2013). Calcium mishandling in diastolic dysfunction: Mechanisms and potential therapies. Biochim. Biophys. Acta.

[60] Guo A., Zhang C., Wei S., Chen B., Song L.S. (2013). Emerging mechanisms of T-tubule remodelling in heart failure. Cardiovasc. Res..

[61] Sutanto H., van Sloun B., Schonleitner P., van Zandvoort M., Antoons G., Heijman J. (2018). The Subcellular Distribution of Ryanodine Receptors and L-Type Ca^2+^ Channels Modulates Ca^2+^-Transient Properties and Spontaneous Ca^2+^-Release Events in Atrial Cardiomyocytes. Front. Physiol..

[62] Koss K.L., Kranias E.G. (1996). Phospholamban: A Prominent Regulator of Myocardial Contractility. Circ. Res..

[63] Gergs U., Mangold W., Langguth F., Hatzfeld M., Hauptmann S., Bushnaq H., Simm A., Silber R.-E., Neumann J. (2019). Alterations of protein expression of phospholamban, ZASP and plakoglobin in human atria in subgroups of seniors. Sci. Rep..

[64] Calloe K., Aistrup G.L., Di Diego J.M., Goodrow R.J., Treat J.A., Cordeiro J.M. (2018). Interventricular differences in sodium current and its potential role in Brugada syndrome. Physiol. Rep..

[65] Wang T., Pang Z.Q., Lin X.Q., Song B.W., Li Z.R., Li S.J., Xia Y.L. (2020). Alterations in the transmural gradient of ventricular repolarization with different pacing sites in normal and heart failure canines. Sheng Li Xue Bao [Acta Physiol. Sin.].

[66] Patel C., Burke J.F., Patel H., Gupta P., Kowey P.R., Antzelevitch C., Yan G.-X. (2009). Is there a significant transmural gradient in repolarization time in the intact heart?. Circ. Arrhythmia Electrophysiol..

[67] Gussak G., Marszalec W., Yoo S., Modi R., O’Callaghan C., Aistrup G.L., Cordeiro J.M., Goodrow R., Kanaporis G., Blatter L.A. (2020). Triggered Ca^2+^ Waves Induce Depolarization of Maximum Diastolic Potential and Action Potential Prolongation in Dog Atrial Myocytes. Circ. Arrhythmia Electrophysiol..

[68] Rice J.J., Jafri M.S., Winslow R.L. (1999). Modeling gain and gradedness of Ca^2+^ release in the functional unit of the cardiac diadic space. Biophys. J..

[69] Patel V.V. (2010). Novel insights into the cellular basis of atrial fibrillation. Expert Rev. Cardiovasc. Ther..

[70] Chen Y.J., Chen S.A. (2007). Thoracic vein arrhythmias. Circ. J. Off. J. Jpn. Circ. Soc..

[71] He B.J., Boyden P., Scheinman M. (2018). Ventricular arrhythmias involving the His-Purkinje system in the structurally abnormal heart. Pacing Clin. Electrophysiol..

[72] Cheniti G., Vlachos K., Meo M., Puyo S., Thompson N., Denis A., Duchateau J., Takigawa M., Martin C., Frontera A. (2018). Mapping and Ablation of Idiopathic Ventricular Fibrillation. Front. Cardiovasc. Med..

[73] Surget E., Cheniti G., Ramirez F.D., Leenhardt A., Nogami A., Gandjbakhch E., Extramiana F., Hidden-Lucet F., Pillois X., Benoist D. (2021). Sex differences in the origin of Purkinje ectopy-initiated idiopathic ventricular fibrillation. Heart Rhythm.

[74] Barber M., Chinitz J., John R. (2020). Arrhythmias from the Right Ventricular Moderator Band: Diagnosis and Management. Arrhythm Electrophysiol. Rev..

[75] Qu Y., Page G., Abi-Gerges N., Miller P.E., Ghetti A., Vargas H.M. (2018). Action Potential Recording and Pro-arrhythmia Risk Analysis in Human Ventricular Trabeculae. Front. Physiol..

[76] van der Weg K., Prinzen F.W., Gorgels A.P.M. (2018). Editor’s Choice- Reperfusion cardiac arrhythmias and their relation to reperfusion-induced cell death. Eur. Heart J. Acute Cardiovasc. Care.

[77] Jongsma H.J., Wilders R. (2000). Gap Junctions in Cardiovascular Disease. Circ. Res..

[78] Guo R., Morimatsu M., Feng T., Lan F., Chang D., Wan F., Ling Y. (2020). Stem cell-derived cell sheet transplantation for heart tissue repair in myocardial infarction. Stem Cell Res. Ther..

[79] Liu M.B., Priori S.G., Qu Z., Weiss J.N. (2020). Stabilizer Cell Gene Therapy: A Less-Is-More Strategy to Prevent Cardiac Arrhythmias. Circ. Arrhythm. Electrophysiol..

[80] Yu J.K., Franceschi W., Huang Q., Pashakhanloo F., Boyle P.M., Trayanova N.A. (2019). A comprehensive, multiscale framework for evaluation of arrhythmias arising from cell therapy in the whole post-myocardial infarcted heart. Sci. Rep..

[81] Khan M., Meduru S., Mohan I.K., Kuppusamy M.L., Wisel S., Kulkarni A., Rivera B.K., Hamlin R.L., Kuppusamy P. (2009). Hyperbaric oxygenation enhances transplanted cell graft and functional recovery in the infarct heart. J. Mol. Cell. Cardiol..

[82] Gratz D., Onal B., Dalic A., Hund T.J. (2018). Synchronization of Pacemaking in the Sinoatrial Node: A Mathematical Modeling Study. Front. Phys..

[83] Greene D.A., Kaboudian A., Wasserstrom J.A., Fenton F., Shiferaw Y. (2022). Voltage mediated mechanism for calcium wave synchronization and arrhythmogenesis in atrial tissue. Biophys. J..

[84] Xie L.H., Sato D., Garfinkel A., Qu Z., Weiss J.N. (2008). Intracellular Ca alternans: Coordinated regulation by sarcoplasmic reticulum release, uptake, and leak. Biophys. J..

[85] Wilhelms M., Hettmann H., Maleckar M.M., Koivumäki J.T., Dössel O., Seemann G. (2012). Benchmarking electrophysiological models of human atrial myocytes. Front. Physiol..

[86] Irvine L.A., Jafri M.S., Winslow R.L. (1999). Cardiac sodium channel Markov model with temperature dependence and recovery from inactivation. Biophys. J..

[87] Beaumont J., Davidenko N., Davidenko J.M., Jalife J. (1998). Spiral Waves in Two-Dimensional Models of Ventricular Muscle: Formation of a Stationary Core. Biophys. J..

[88] Sutanto H. (2022). Individual Contributions of Cardiac Ion Channels on Atrial Repolarization and Reentrant Waves: A Multiscale In-Silico Study. J. Cardiovasc. Dev. Dis..

