# Supplementary material for: Critical Requirements for the Initiation of a Cardiac Arrhythmia in Rat Ventricle: How Many Myocytes?
Source: Cells. 2022 Jun 9;11(12):1878. doi: 10.3390/cells11121878 (PMC9221049; doi:10.3390/cells11121878)
Supplement: Supplementary file 1 [file cells-11-01878-s001.zip › Supplementary Caption.pdf]

Video S1 – Simulated action potential propagation across normal ventricular myocytes in a 100 x 100 network initiated by current injection.

Video S2 – Simulated action potential triggered calcium release across normal ventricular myocytes in a 100 x 100 network initiated by current injection.

Video S3 – Simulated action potential propagation across failing ventricular myocytes in a 100 x 100 network initiated by current injection.

Video S4 – Simulated action potential triggered calcium release across failing ventricular myocytes in a 100 x 100 network initiated by current injection.

Video S5 – Simulated action potential propagation across normal ventricular myocytes in a simulated trabeculae connected to the ventricular wall initiated by current injection.

Video S6 – Simulated action potential propagation across failing ventricular myocytes in a simulated trabeculae connected to the ventricular wall initiated by current injection.
